# Assessing Adherence to Cardiac Monitoring Guidelines in Trastuzumab-Treated Breast Cancer Patients: Insights From a Tertiary Hospital

**DOI:** 10.7759/cureus.48832

**Published:** 2023-11-15

**Authors:** Abeer Alhuthali, Abdullah Alshammari, Khaldoun Saleh, Mohammed Jaffal, Eshtyag Bajnaid, Masaad S Almutairi, Ziyad Almuylibi, Alanoud Alghanmi, Mohammed Alnuhait

**Affiliations:** 1 Department of Clinical Pharmacy, King Abdullah Medical City, Makkah, SAU; 2 Department of Pharmacy Practice, College of Pharmacy, Umm Al-Qura University, Makkah, SAU; 3 Department of Pharmacy Practice, Qassim University, Buraydah, SAU; 4 Department of Pharmacy, King Fahad Hospital, Ministry of Health, Al-Baha, SAU; 5 Department of Pharmacy, King Fahad Armed Forces Hospital, Jeddah, SAU

**Keywords:** adherence, guidelines, breast cancer, cardiac, trastuzumab

## Abstract

Introduction: Breast cancer is a global health concern, with a significant portion of patients exhibiting human epidermal growth factor receptor 2 (HER2) overexpression. Trastuzumab is one of the pivotal therapies for HER2-positive breast cancer, but it carries the risk of cardiotoxicity. Guidelines for cardiac monitoring are essential to detect early signs of cardiotoxicity. However, adherence to these guidelines remains uncertain.

Method: In this single-center retrospective cohort study, we analyzed data from 167 female patients diagnosed with HER2-positive breast cancer who were treated with trastuzumab. We meticulously assessed the level of adherence to cardiac monitoring guidelines and determined the incidence of trastuzumab-induced cardiotoxicity (TIC). Factors affecting adherence were subsequently investigated using appropriate statistical methods.

Results: Adherence to monitoring guidelines was only 31.7%. TIC incidence was 7.8%. Patients with concurrent use of cardiotoxic medications demonstrated higher adherence. A significant association was found between the number of trastuzumab doses and adherence.

Conclusion: Adherence to monitoring guidelines was suboptimal. Those at a higher risk of cardiac issues showed greater adherence. Improved risk assessment methods are needed to individualize monitoring and intervention. Future research should focus on patient-centered, evidence-based monitoring to optimize the balance between cancer therapy and cardiac safety in the field of cardio-oncology.

## Introduction

Breast cancer is a prevalent global health issue that affects primarily female populations, thus constituting a significant public health concern with far-reaching implications within the field of oncology [1,2]. Available data show that over 3.8 million women in the United States (USA) have a documented history of breast cancer [3,4]. The American Cancer Society reported that, in 2022, there were 297,790 new cases of invasive breast cancer identified in women [5]. In particular, approximately one-quarter of breast cancer cases manifest overexpression of the human epidermal growth factor receptor 2 (HER2) [1,6]. HER2 plays a crucial role in the normal growth and development of various tissues, and its overexpression precipitates increased cell proliferation and an increased presence of malignant cells [7]. Consequently, trastuzumab, a humanized monoclonal antibody designed to target HER2, exerts its therapeutic effect by inhibiting cell proliferation and inducing cell apoptosis. Consequently, the use of trastuzumab in patients with HER2-positive breast cancer assumes a central role in improving survival rates. Trastuzumab is recommended as an adjunctive therapy in conjunction with chemotherapy for patients with breast cancer who exhibit HER2 overexpression. This combined approach underscores the importance of trastuzumab in the comprehensive management of HER2-positive breast cancer cases [8-10]. Indeed, it should be noted that HER2 is expressed not only in cancer cells but also in the myocardium, specifically within the myocytes of the heart. Therapeutic use of trastuzumab is associated with an elevated risk of cardiovascular complications, particularly left ventricular dysfunction. The appearance of trastuzumab-induced cardiac dysfunction varied according to definition and severity [11]. The definition of cardiotoxicity attributed to trastuzumab treatment lacks universal consensus; nevertheless, an acceptable criterion is the reduction of the left ventricular ejection fraction (LVEF) by more than 10 percentage points, resulting in LVEF measurement falling below 50%. This parameter serves as a clinically relevant indicator of cardiac dysfunction in patients undergoing trastuzumab therapy [8,10]. Despite the elevated risk of cardiotoxicity associated with trastuzumab, it is important to recognize that optimal monitoring protocols for this condition have not been definitively established through prospective cohort studies. Consequently, cardiotoxicity monitoring is primarily based on expert consensus and regulatory agencies, such as the Food and Drug Administration (FDA) [8-12]. The FDA, for example, recommends a monitoring schedule that involves cardiac function evaluations every three months during the first year of treatment and then extends to every six months during the second year. This approach aims to detect any early signs of cardiotoxicity and guide clinical decisions accordingly [9]. According to the guidance provided by the National Comprehensive Cancer Network (NCCN), it is recommended to implement structured cardiac monitoring at designated intervals throughout the duration of trastuzumab therapy. These monitoring intervals are consistent with the recommendations set forth by the FDA [10].

This underscores the need for careful patient selection and stringent monitoring when using trastuzumab as a therapeutic measure. Our study is pivotal as it pioneers the effort to link cardiac toxicity with adherence to prescribed cardiac monitoring guidelines. We focus on patients diagnosed with HER2-positive breast cancer undergoing trastuzumab treatment. By delving into the correlation between compliance with monitoring protocols and cardiac toxicity occurrence, our study seeks to bridge a vital knowledge chasm in the discipline. Fundamentally, our findings hold the promise to enhance clinical practice, enriching the care quality for this distinct patient group.

## Materials and methods

Method

This retrospective cohort study was conducted in a single center and focused on patients diagnosed with HER2-positive breast cancer who received trastuzumab as part of adjuvant or metastatic treatment protocols during the period of June 1, 2019, to June 1, 2020. To maintain the integrity of the study, people with a primary diagnosis of another type of cancer or those who lost follow-up were excluded from the analysis. Patient data were collected from the electronic health system, which includes information related to patient characteristics, comorbidities, and the concurrent administration of cardiotoxic medications. The primary objective of this investigation was to evaluate the adherence rate to cardiac monitoring guidelines among the patient cohort. Adherence was operationally defined as the achievement of 75% compliance with cardiac monitoring, which encompassed cardiac function assessments at baseline and at least twice during therapy for one year. The secondary objective of this study was to determine the incidence of trastuzumab-induced cardiotoxicity (TIC). TIC was defined as symptomatic heart failure or an asymptomatic decline in left ventricular ejection fraction (LVEF) that is 10% or more, or an LVEF value below or equal to 50% using echocardiography. Another objective is to evaluate the elements that contributed to enhanced compliance with the guideline recommendations. Echocardiography is a non-invasive diagnostic tool that uses sound waves to produce detailed images of the heart's structure and function. In the context of trastuzumab therapy, regular echocardiography tests are crucial given trastuzumab's potential cardiotoxic effects. Patients undergoing trastuzumab treatment for HER2-positive breast cancer are at an elevated risk for cardiac dysfunction, specifically a reduction in LVEF. As such, an echo test is performed to establish a baseline LVEF prior to initiating therapy. Subsequent echocardiographic evaluations are then routinely scheduled to monitor for any decline in LVEF or other cardiac abnormalities during treatment. Early detection of cardiac changes via echo allows clinicians to make timely and informed decisions, such as modifying trastuzumab dosage or temporarily halting its administration.

The study received approval from the Institutional Review Board of King Abdullah Medical City, Makkah, Saudi Arabia, to ensure ethical compliance in conducting this research (Registration No. H-02-K-001).

Statistical analysis

Continuous variables were displayed as means with their corresponding standard deviations, while categorical data were represented using frequency and percentage distributions. A descriptive analysis was performed to assess the degree of adherence to the cardiac monitoring regimen among patients with HER-2-positive status who were on trastuzumab therapy. Additionally, the chi-square method was used to investigate possible connections between adherence to monitoring and hospital admissions related to cardiac toxicity during treatment. Furthermore, an independent t-test was used to clarify the relationship between the number of doses received and adherence to established clinical guidelines.

## Results

**Figure 1:**
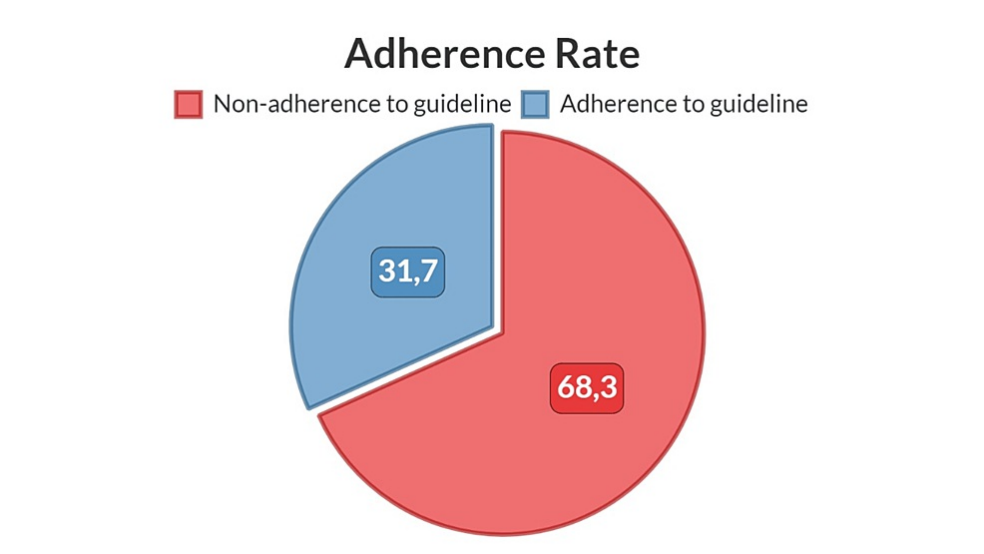
Percentage of compliance with echocardiography monitoring guidelines.

The study involved a cohort of 167 female patients, all diagnosed with breast cancer. The average age of these participants is 52 years. The majority of patients exhibited no prior history of heart failure, with two notable exceptions. Hypertension was diagnosed in 15% of the cohort. Noteworthily, a significant 92.8% underwent concurrent trastuzumab and standard chemotherapy. A notable 57% administered trastuzumab alongside drugs with potential cardiotoxic implications. Table 1 elaborates on the baseline characteristics of the study cohort. Central to the study's objective, a 31.7% adherence rate to echocardiographic monitoring protocols was recorded, as visually represented in Figure 1. The incidence of TIC was 7.8%. Of the 12 individuals who manifested cardiotoxicity, seven followed the guidelines. Out of the 167 individuals studied, 155 (92.8%) were administered concurrent chemotherapy. When divided based on adherence to guidelines, 105 (73%) of the non-adherence group and 50 (94.3%) of the adherence group underwent concurrent chemotherapy. Furthermore, 96 participants (57.5%) received concurrent cardiotoxic chemotherapies. Of these, 52 (54.2%) were non-adherent to guidelines, and 44 (45.9%) adhered to the guidelines. The observed difference in the use of cardiotoxic chemotherapies between the two groups was statistically significant, with a p-value of 0.001. Regarding the outcomes, the average number of cycles administered was 12 ± 7.1. The non-adherence group had a slightly lower mean of 11.7 ± 7.5 cycles, compared to the adherence group which had a mean of 14.9 ± 5.7 cycles. This difference was statistically significant with a p-value of 0.005. Finally, 12 individuals (7.18% of the total cohort) were admitted due to cardiotoxicity. Out of these, five were from the non-adherence group, and seven were from the adherence group.

**Table 1:** Baseline characteristics.

Baseline characteristics	Mean ± SD
Age, years	52.4 ± 11.07
Weight, kg	69.24 ± 16.73
	N (%)
Gender, Female	167 (100%)
Comorbidities	
Preexisting heart failure	2 (1.2 %)
Hypertension	25 (15 %)
Others	17 (10.2 %)
Medications	
Concurrent chemotherapy	155 (92.8 %)
Concurrent cardiotoxic chemotherapies	96 (57.5 %)

## Discussion

The advent of anti-HER2 targeted therapy has marked a pivotal shift in the management of HER2-positive breast cancer, significantly enhancing patient outcomes. Nonetheless, these therapeutic strides come with challenges, particularly as treatments based on trastuzumab have been associated with increased cardiac toxicity risks [8,9,13]. This discussion aims to contextualize our research findings, focusing on adherence to monitoring guidelines, the emergence of cardiotoxicity, and the pressing demand for precise risk assessment tools.

Alarmingly, our study highlighted a pronounced non-adherence to the monitoring guidelines. Merely 31.7% of patients conformed to the suggested cardiac surveillance protocols. This diminished adherence, which mirrors the findings from earlier research, is particularly worrisome given the established risks of trastuzumab-based regimens [7,14,15]. However, our data reported a cardiotoxicity rate of only 7.8%. This rate is congruent with many preceding studies that have recorded cardiotoxicity rates between 1.4% and 27%, contingent on the severity gradient and the investigated patient demographics [4,8,14,16]. This underscores the urgency to establish dependable prediction tools, such as risk stratification nomograms, to pinpoint individuals with heightened cardiotoxicity risks. Such methodologies can pave the way for rigorous monitoring of high-risk individuals, facilitating timely interventions.

Notably, our study unveiled that patients with elevated cardiac risks exhibited better adherence to the prescribed monitoring protocols. The data suggest that individuals concurrently taking cardiotoxic drugs and undergoing more extensive trastuzumab therapy were more inclined to rigorously adhere to monitoring directives. This observation resonates with findings from Rushton et al. and accentuates the importance of stringent monitoring among groups with augmented cardiotoxicity risks [9].

Given the occasional and generally reversible nature of cardiotoxicity, there is a resounding call for enhanced cardiac surveillance [8,15,17] Nonetheless, mandating frequent cardiac monitoring for those with a lower risk profile could inflate healthcare costs, especially due to the steep prices of imaging diagnostics [7,9].

These insights spotlight existing knowledge lacunae in cardio-oncology. A universally standardized cardiotoxicity screening method might prove inadequate [7,17]. The focus ought to shift toward pinpointing specific cohorts with pronounced cardiotoxicity risks. Past studies have elucidated several predictors of cardiotoxicity, including age above 50, compromised baseline LVEF, historical hypertension, and prior anthracycline exposure [8,14,17]. However, the reliability of these risk markers demands rigorous validation.

To enrich our understanding of optimal monitoring schemes for high-risk categories, it is imperative to commission randomized trials or prospective registries [9]. These endeavors should elucidate monitoring outcomes and vouch for the merits of early intervention modalities [4,8,10,14]. Advancements in cardio-oncology hinge on addressing these focal areas, culminating in a patient-centric, risk-adjusted approach for cardiotoxicity surveillance. This would synergize efficacious cancer therapy with cardiac safety.

Limitations of the study

While our research emphasized more frequent monitoring, especially among high-risk individuals, the potential economic implications of this strategy were not deeply explored. The study suggests that a standardized cardiotoxicity screening might not be universally applicable. Although various cardiotoxicity predictors have been identified, their definitive reliability remains uncertain. Our findings stress the need for further research, including randomized trials, to ensure effective cancer treatment that balances cardiac safety. Our study revealed substantial non-compliance with cardiac monitoring guidelines, emphasizing the need for precise assessment tools. Future directions should prioritize developing predictive tools, understanding optimal monitoring for high-risk groups through randomized trials or broad registries, and promoting tailored, patient-centric monitoring strategies.

## Conclusions

The study illuminates significant non-adherence to cardiac monitoring guidelines among HER2-positive breast cancer patients on trastuzumab therapy, despite known cardiotoxicity risks. While overall cardiotoxicity rates were relatively low, there is a pressing need for precise risk assessment tools and tailored monitoring approaches. Emphasizing patient-specific, risk-adjusted strategies will harmonize effective cancer treatment with cardiac safety, driving advancements in cardio-oncology.
